# Protocol for determining the diagnostic validity of physical examination maneuvers for shoulder pathology

**DOI:** 10.1186/1471-2474-14-60

**Published:** 2013-02-08

**Authors:** Lyndsay Somerville, Dianne Bryant, Kevin Willits, Andrew Johnson

**Affiliations:** 1 School of Physical Therapy, Faculty of Health Sciences, Western University, London, Canada; 2 Health and Rehabilitation Sciences, Faculty of Health Science, Western University, London, Canada; 3 Department of Clinical Epidemiology & Biostatistics, Faculty of Health Sciences, McMaster University, Hamilton, Canada; 4 Division of Orthopaedic Surgery, Schulich School of Medicine & Dentistry, Western University, London, Canada; 5 School of Health Studies, Faculty of Health Sciences, Western University, London, Canada

## Abstract

**Background:**

Shoulder complaints are the third most common musculoskeletal problem in the general population. There are an abundance of physical examination maneuvers for diagnosing shoulder pathology. The validity of these maneuvers has not been adequately addressed. We propose a large Phase III study to investigate the accuracy of these tests in an orthopaedic setting.

**Methods:**

We will recruit consecutive new shoulder patients who are referred to two tertiary orthopaedic clinics. We will select which physical examination tests to include using a modified Delphi process. The physician will take a thorough history from the patient and indicate their certainty about each possible diagnosis (certain the diagnosis is absent, present or requires further testing). The clinician will only perform the physical examination maneuvers for diagnoses where uncertainty remains. We will consider arthroscopy the reference standard for patients who undergo surgery within 8 months of physical examination and magnetic resonance imaging with arthrogram for patients who do not. We will calculate the sensitivity, specificity and positive and negative likelihood ratios and investigate whether combinations of the top tests provide stronger predictions of the presence or absence of disease.

**Discussion:**

There are several considerations when performing a diagnostic study to ensure that the results are applicable in a clinical setting. These include, 1) including a representative sample, 2) selecting an appropriate reference standard, 3) avoiding verification bias, 4) blinding the interpreters of the physical examination tests to the interpretation of the gold standard and, 5) blinding the interpreters of the gold standard to the interpretation of the physical examination tests. The results of this study will inform clinicians of which tests, or combination of tests, successfully reduce diagnostic uncertainty, which tests are misleading and how physical examination may affect the magnitude of the confidence the clinician feels about their diagnosis. The results of this study may reduce the number of costly and invasive imaging studies (MRI, CT or arthrography) that are requisitioned when uncertainty about diagnosis remains following history and physical exam. We also hope to reduce the variability between specialists in which maneuvers are used during physical examination and how they are used, all of which will assist in improving consistency of care between centres.

## Background

According to Sackett and Haynes [1] studies evaluating the diagnostic validity of clinical tests are classified along a continuum from efficacy (Phase I and Phase II) to effectiveness (Phase III and Phase IV). Whereas efficacy studies offer information about diagnostic validity under ideal conditions (i.e. disease status is known) effectiveness studies, offer practical information about the validity of the diagnostic test under usual conditions in a clinical setting [1]. Because the diagnosis is known in efficacy studies, their results are not applicable to clinical settings where patients’ diagnoses are unknown until after the tests are completed and their results interpreted.

Shoulder complaints are the third most common musculoskeletal problem in the general population, and are second only to knee pain referrals to orthopaedic surgery or primary care sports medicine [2]. Patients who present with shoulder pain pose diagnostic challenges for physicians due to the numerous pathologies and the potential for multiple disorders to exist within the same patient.

Most physicians rely on a thorough history to aid in the diagnosis of shoulder pain and in fact, the history is a diagnostic test itself; however, to date few studies have evaluated the accuracy of the history as a diagnostic test. One example by Litaker et al. [3], retrospectively assessed 448 patients suspected of having rotator cuff disease who underwent magnetic resonance arthrography as the reference standard. They evaluated the ability of items from the patient history to diagnose rotator cuff tears. They demonstrated that a history of trauma is not sensitive (36%) in diagnosing rotator cuff tears however it is relatively specific (73%) when the patient does not have a rotator cuff tear. In addition they found that night pain was highly sensitive (87.7%) for diagnosing rotator cuff tears.

Three important aspects of the history are the presence or absence of certain symptoms, the duration of symptoms and the mechanism of injury. For example, pain characteristics such as location, quality, radiation, and aggravating and/or relieving factors are helpful in diagnosing the source of shoulder pain and/or disability. Longer symptom duration may indicate an overuse injury, such as tendinosis, whereas an acute onset of symptoms may be indicative of an acute or traumatic injury, such as shoulder dislocation. The mechanism of injury can differentiate between competing diagnoses like anterior versus posterior instability, or SLAP versus rotator cuff tear. A comprehensive history, however, also includes characteristics of the patient. For example, since the incidence of rotator cuff pathology increases with age [4,5], age is an important component of the history.

In addition to the history, an abundance of physical examination maneuvers have been developed for diagnosing shoulder pathology. These maneuvers are a common component in establishing a diagnosis and determining a treatment plan however, the accuracy of many of these tests has not been adequately addressed. Several systematic reviews [6-10] have noted a lack of methodological quality in studies reporting the accuracy of physical exam maneuvers for diagnosing shoulder pathology. The most recent reviews [9,10] have argued for the need for large, well designed studies that examine the accuracy of numerous physical examination tests for the shoulder. We performed a systematic review of the literature to determine the diagnostic validity of physical examination tests for shoulder pathology, including rotator cuff disease, labral lesions, instability and acromioclavicular joint abnormalities [11]. The initial search strategy yielded a total of 5977 potentially relevant studies (336 CINAHL, 922 EMBASE, 4719 MEDLINE). Initial screening of titles and abstracts reduced this to 333 articles. Agreement between reviewers was good (κ = 0.71, SE = 0.01). Following formal full text review, 49 studies remained eligible. Agreement between reviewers was excellent (κ = 0.95, SE = 0.03). We identified one unpublished abstract from the conference proceedings of the annual American Association of Orthopaedic Surgeons (AAOS). Our secondary search of reference lists yielded one unpublished abstract and four eligible studies. Eligible studies were defined according to design (efficacy versus effectiveness) as Phase I (efficacy), Phase II (efficacy), or Phase III (effectiveness), and were also reviewed for methodological quality.

A total of 55 studies were eligible. Only 2% (1/55) of the studies we identified were classified as Phase III studies. None of the included studies met all of the quality criteria. We argue that in addition to the need for high quality studies proposed by others, there is a need to better understand the difference between efficacy and effectiveness studies, when each study design is justified and what they can and cannot offer in terms of applicability to a clinical setting.

The purpose of this paper is to present a proposal for a prospective study to evaluate the diagnostic validity of clinical examination tests for common disorders of the shoulder including rotator cuff pathology, labral pathology, and instability.

## Methods

### Objectives

#### History

1) To determine the sensitivity, specificity and positive and negative likelihood ratio of patient reported history items for shoulder pathology including items for rotator cuff pathology, labral pathology (SLAP, instability and other labral lesions), and AC joint pathology.

2) To identify which patient reported history items best predict each of the disease states. We will then determine the top items for each disease state.

3) To identify how often physicians are correct in their diagnosis following history alone. Additionally we will determine if the physical examination adds to the clinicians’ confidence in their diagnosis made by the history alone.

#### Physical examination

1) To determine the sensitivity, specificity, and positive and negative likelihood ratio of all included physical examination tests for shoulder pathology including tests for rotator cuff pathology, labral pathology (SLAP, instability and other labral lesions), and AC joint pathology.

2) To identify the top physical examination maneuvers for each disease state.

3) To determine the likelihood ratio for different combinations of tests for each disease state and to make a recommendation to clinicians as to the combination of tests that are most valid (i.e. reduce physician uncertainty) in establishing a diagnosis.

### Study design and setting

**Figure 1:**
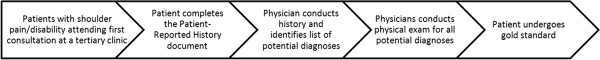
Flow diagram.

We will conduct a prospective cohort study recruiting consecutive new patients who present with shoulder pain to the Fowler Kennedy Sport Medicine Clinic, London Health Sciences Centre (University Campus) or to St. Joseph’s Healthcare in Hamilton, Ontario, Canada (see Figure 1). Each participating physician will identify potentially eligible patients to the research assistant who will describe the study to the patient and provide a written Letter of Information and Consent when the patient arrives for their first consultation. This study was approved by the Health Sciences Research Ethics Board at Western and McMaster University, in Ontario, Canada.

**Figure 2:**
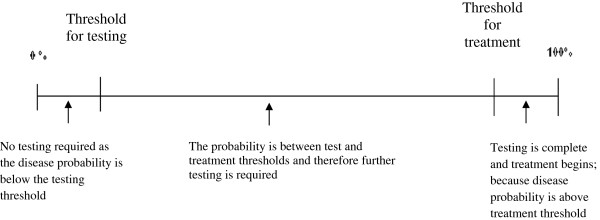
Treatment and testing thresholds in the diagnostic process.

The sample of patients selected for study participation must be representative of the population of patients with shoulder complaints for which the physician would face diagnostic uncertainty in a typical orthopaedic practice. This includes patients who have a variety of diagnoses that represent the full spectrum of what would usually be seen in a typical practice, including patients with and without concomitant pathology and those with other shoulder pathology that present with similar symptoms. One method to ensure that a representative sample is included is to recruit consecutive patients. Thus, our approach to sampling will include recruiting all new patients with shoulder complaints who are scheduled for their first consultation with an orthopaedic surgeon. Upon taking a history (and without review of any prior imaging or tests), the surgeon will provide the pre-test probability for eight possible diagnoses (see Figure 2).

### Selection of physical examination tests

We identified all physical examination tests through a systematic review of the literature. This process identified 74 physical examination tests for shoulder pathology. We used a modified Delphi process to determine which of these 74 physical examination tests to include in our study. To do this, we administered an online survey, using Survey Monkey (©2005 SurveyMonkey.com), to the five participating surgeons with expertise in shoulder physical examination and surgery who were asked to identify their preference to include or exclude each test. The survey included the original description of the test and any subsequent modifications along with the original and modified instructions for scoring each test. Next, we tallied the results of this survey and included tests for which the majority of surgeons indicated that the test should be included, excluded tests for which the majority of surgeons indicate that the test should not be included, and produced a second survey for tests for which no majority was reached. In each case, ‘majority’ was defined as at least 4 of 5 surgeons.

All surgeons completed all rounds of the Delphi process. Following the first round of the modified Delphi survey, 14 tests were marked as include and 28 tests were marked as exclude. There was a discrepancy for 32 tests; these were included in the second survey.

The second survey presented the results of the first survey and identified tests for which there were discrepancies between surgeons. This survey asked each surgeon to present arguments for why the test should or should not be included in the study and to reaffirm their decision. If, following this second survey, any test was still without a majority decision, a document reproducing the argument for and against including each test was created and circulated, and a meeting with the surgeons was held until consensus was reached.

Following the second survey, there were 11 tests without a majority decision. Following the third survey round where surgeons provided free-text arguments for or against the inclusion of the remaining 11 tests and a revote, consensus was reached; nine tests were included and two were excluded. Therefore, a total of 32 tests will be included in the study. Included tests are presented in Table 1 by shoulder pathology.

**Table 1:** Included tests

**General**	**Rotator cuff pathology**	**Labral lesions**	**Instability**	**AC abnormalities**
**ROM**	**General**	**SLAP**	**Anterior**	O’Briens Test
Forward Flexion	Transdeltoid Palpation	Speeds Test	Load and Shift	Cross Body Adduction Stress Test
External Rotation	**Tendinosis**	Anterior Slide Test	Apprehension Test	
Internal Rotation	Painful Arc	Active Compression	Relocation Test	
**Strength**	Hawkins Kennedy	Compression Rotation	Surprise/Release Test	
External Rotation	Neers Impingement	Biceps Load Test I	**Posterior**	
Internal Rotation	**Supraspinatus**	Biceps Load Test II	Posterior Apprehension	
	Jobes Test	Resisted Supination External Rotation	Modified Barlow Test	
	Full Can Test	**Other Labral**	**Multidirectional**	
	**Infraspinatus**	Kims Test	Sulcus Sign	
	External Rotation Lag			
	**Subscapularis**			
	Lift Off Test			
	Belly Press Test			
	Internal Rotation Lag			

### Clinical examination testing

Richardson, Wilson and Guyatt [12] have identified two underlying steps to differential diagnosis. The first step involves arriving at a list of diagnostic possibilities and their relative likelihood of being responsible for the patient’s complaints. The first attempt at listing the possible diagnoses comes from listening to the patient describe the history behind the symptoms. The relative likelihood, coined the *pretest probability*, is the probability that the patient has the disease of interest based on the physician’s experience with the disease and the signs and symptoms presented by the patient [12].

In the second step, diagnostic tests are performed or administered by the physician and the results of those tests are used to revise the initial pretest probability to a *posttest probability*. The posttest probability is the probability that the patient has the disease of interest following the results of a diagnostic test [12]. It follows then that the diagnostic process involves a continuum of probabilities between two thresholds (Figure 2); where a probability of 0.50 or 50:50 chance of having the disease represents the greatest amount of uncertainty, probabilities less than 0.50 indicate greater certainty that the disease is not the cause of the patient’s symptoms, and probabilities greater than 0.50 indicate greater certainty that the disease is contributing to the symptoms. In fact, the clinician’s perception about the probability of having a specific disease may become sufficiently high that it surpasses the *treatment threshold*, such that the physician recommends therapy without further testing. On the other hand, the clinician’s perception about the probability of having a particular disease may become sufficiently low that it falls below the *test threshold*; at which point no further testing is recommended and the clinician rules out the disease.

The more accurate the diagnostic test, the greater the reduction in uncertainty about the diagnosis either toward dismissing a particular diagnosis from the list of possibilities or toward offering treatment for a highly probable disease. Less powerful diagnostic tests are unlikely to sufficiently change the degree of uncertainty, sometimes necessitating more invasive or expensive tests to further reduce uncertainty and reach a final diagnosis. For example, if physical examination tests cannot differentiate between a significant SLAP lesion and a rotator cuff tear, the surgeon whose expertise is insufficient to perform an arthroscopic SLAP repair has essentially just performed a risky, invasive and expensive diagnostic test by performing the arthroscopic examination without being able to offer treatment.

In our study, therefore, the physician will take a thorough history including, mechanism of injury, duration of symptoms, history of shoulder injuries and patient characteristics such as age, occupation and daily activities. Following the history, the physician will indicate the pretest probability of eight common shoulder pathologies using a 100 mm visual analogue scale (VAS). These will include rotator cuff tendinopathy, rotator cuff tear, AC joint pathology, SLAP lesion, other labral lesions and instability (anterior, posterior, or multi-directional each represented by a separate scale).

Patients for whom the physician feels some uncertainty in the diagnosis (i.e. placed a mark between the two thresholds) will undergo the physical examination tests for those diagnoses only. For example, if the physician is certain that the patient has instability without AC joint pathology, though he or she remains uncertain about the direction of instability, this patient will undergo physical examination tests for instability but will not undergo the tests for AC joint pathologies. Similarly, the clinician may be certain that the patient does not have instability (i.e. the pretest probability that the patient has instability is below the testing threshold) but is uncertain whether the diagnosis is tendinosis or more severe rotator cuff pathology, a labral lesion or AC joint pathology. This patient would undergo physical examination tests for tendinosis, rotator cuff tears, labral lesions and AC joint osteoarthritis but tests for instability would not be performed.

To standardize the technique and scoring for each test, we constructed a glossary (Additional file 1) that will be provided to clinicians. Each clinician is required to review the glossary and ensure their method of application matched the description provided.

To assist with standardization, we included pictures that illustrate the technique. Further, we will use a standardized data collection form that includes the description of how each test is performed and scored. Finally, the graduate student will be trained how to perform all physical examination tests and familiarized with alternative techniques so that she can provide correction if the clinician is performing the test in a manner other than as described in the protocol. Tests will be ordered according to the position of the patient during the test (e.g. seated, supine, standing) although the clinician will be free to order the tests as he or she sees fit. A research assistant will be present to ensure that all tests are completed and to record the results of the test on the data collection form.

The research assistant will remove any imaging studies, reports or other test results from the patient’s chart so that the clinician performing the tests is not biased in their interpretation of the physical examination tests. All imaging and other tests including any reports will be made available to the clinician after the physical examination tests are complete.

### Choice of reference standards

One of the most common methodological flaws within the literature of diagnostic validity studies for shoulder physical examination tests is the exclusion of patients who did not undergo surgery. Obviously not all patients who present to an orthopaedic practice are recommended for surgery or elect to undergo recommended surgery. The sample formed by excluding these two subpopulations from the greater population of patients with shoulder pain or disability is no longer representative of typical clinical practice. Further, we might expect that estimates of the accuracy of physical examination tests that are restricted to patients who ultimately undergo surgical treatment are overly optimistic since the sample is made up of a non-representative proportion of (perhaps) more severely affected individuals.

Thus, this study will include two comparable reference standards. We will use arthroscopic examination as the reference standard for patients who undergo surgical treatment within eight months of physical examination, and magnetic resonance imaging with arthrogram (MRA) for patients who do not undergo surgery within this timeframe.

We developed a standardized arthroscopic examination and reporting protocol to minimize differences between surgeons in diagnoses due to variations in methods of examination (Additional file 2) and to minimize any detection bias should the clinician recall the physical examination or results of imaging or other special tests at the time of interpreting the surgical examination.

MRA was chosen as the reference standard over MRI due to its ability to diagnose disorders of the internal soft tissue structures such as the labrum. The literature has shown that MRI is not as accurate for diagnosing SLAP tears as MRA with reported sensitivities for MRI ranging from 43% - 75% [13-17] and specificities between 58% - 70% [14,15,17]. MRA has been shown to be highly sensitive and specific for detecting both rotator cuff pathology and labral injuries [18,19]. In some cases patients will undergo both surgery and an MRA. For these cases we will calculate the agreement between these two standards to further justify the use of MRA as a second reference standard.

### Plan for statistical analyses

We will calculate sensitivity and specificity for each test individually including 95% confidence intervals around these estimates. Sensitivity is calculated by dividing the number of patients with the disease who had a positive test (true positive) by the total number of patients with the disease. Specificity is calculated by dividing those without the disease who had a negative test (true negative) by the total number of patients without the disease. We will use these values to calculate positive and negative likelihood ratios (LR). A positive likelihood ratio is the likelihood that a positive test result is elicited in a patient with the target disorder compared to the likelihood that a positive test result is elicited in a patient without the target disorder (sensitivity/(1-specificity)). A negative likelihood ratio is the likelihood that a negative test result is elicited in a patient with the target disorder compared to the likelihood that a negative test result is elicited in a patient without the target disorder ((1 – sensitivity)/specificity). LRs have advantages over sensitivity and specificity because they can be calculated for several levels of the symptom/sign or test, they can be used to combine the results of multiple diagnostic test and they can be used to estimate a post-test probability for a target disorder all of which is more useful in a clinical setting.

We will divide the tests into groups according to which disease they tested for. We will then dummy code these sets of tests to indicate whether one test, two tests or all tests are positive. We will test whether combinations of the tests improves the ability to diagnose disease. We will calculate the sensitivity, specificity and likelihood ratio if all tests positive, one test is positive, at least one test is positive and so on. Additionally we will assess whether particular tests can be removed from the set of tests without losing any diagnostic ability for each disease. Poor indicators of disease will be removed from the analysis and the change in accuracy measures will be evaluated. This analysis will determine the appropriate number and combinations of tests for each disease category that will provide the greatest clinical yield.

### Estimation of sample size

To address our first two hypotheses, we assumed a sensitivity and specificity of at least 0.85 with a 95% confidence interval with a bounds of +/− 0.10. This boundary was selected because the authors felt that if the uncertainty around the estimate of validity included the possibility of a sensitivity or specificity of less than 0.75 that the conclusions about the usefulness of the test change. Using these parameters a sample size of 50 patients tested at each disease state (AC joint pathology, rotator cuff pathology, SLAP lesions, other labral lesions, and anterior instability) is required [20]. Since some of these patients may be lost-to-follow-up or drop out, we inflated this sample size by 10% for a total of 55 patients tested in each disease category.

Since maintaining the distribution of disease severity is crucial to the validity of our study, we will recruit consecutive patients up to and until the required 55 patients are recruited for the slowest recruiting disease category. We anticipate that some patients will have multiple diagnoses (e.g. rotator cuff tear and SLAP lesion), which will mean that they are counted as disease positive for more than one analysis, thus our sample size for each disease group is likely to be larger than the required 55 patients tested per disease group.

### Steps taken to minimize bias

We have taken the following 4 steps to minimize bias in our study,

1) Minimization of Disease Progression Bias

Disease progression bias occurs when the time between administration of the reference standard and the physical examination maneuver is such that the disease of interest has changed [21]. To avoid disease progression bias, any patient not undergoing surgery within 8 months of physical examination will undergo an MRA of the affected shoulder. Several studies have demonstrated that rotator cuff tears can progress over time [22,23]. However, both Safran et al. [22] and Yamaguchi demonstrated that only 50% of their sample had an increase in tear size at greater than 2 years follow-up. Therefore orthopaedic clinicians with a specialty in shoulder surgery chose 8 months as a time point they felt was reasonable where disease would not change from the time of initial consult. In these cases, the MRA will serve as the reference standard.

2) Minimization of Interpretation Bias

Interpretation bias may be present if the results of the test are known by the individual responsible for interpreting the reference standard or vice versa. As this is a prospective study, the natural order ensures that clinicians are unaware of what will be found during surgical examination at the time they perform and interpret physical examination tests. Since experience is an important influence on how physical examination tests are performed and interpreted [24-26], the consultant will perform both the physical examination tests and the surgery. Although this prevents outright blinding of the clinician to the results of the physical examination at the time they are performing and interpreting the arthroscopic examination, the volume of patients participating in this study and the time between physical examination and surgery will reduce the likelihood that clinicians will recall the results of the physical examination. Clinicians were not permitted to repeat any component of the physical examination prior to the surgery. Further, we standardized the arthroscopic examination to avoid biased approaches to the examination (i.e. close examination of the suspected source of the problem and little or no examination of other structures). Finally, a radiologist with expertise in musculoskeletal imaging who is blind to the results of the physical examination and to other imaging results or reports will interpret the MRA of patients who do not undergo surgical examination.

3) Ensuring a Representative Sample

We will take three steps to ensure the representativeness of our sample. First, we will recruit patients consecutively from the practices of three orthopaedic surgeons at different stages of practice (>15 years, >6 years and <5 years). Second, following a thorough history, we will assess diagnostic uncertainty for each of the common shoulder disorders by recording the degree of certainty (or uncertainty) using a figure similar to Figure 1 for each disease, and similar to usual practice, the clinician will only perform physical examination tests for diseases that the clinician feels are possible explanations for the patient’s complaints. Third, the entire sample of patients will undergo a reference standard – either surgery or MRA since to include only those patients who undergo surgery is to include those more likely to test positive on a physical examination test, providing an overestimate the sensitivity of the test.

4) Avoidance of Verification Bias

Verification bias occurs when the results of the diagnostic test influence the clinician’s decision as to which patients undergo the gold standard. We also wish to emphasize that verification bias is also probable if it is some other test (not the test under evaluation) that influences the clinician’s decision to recommend the gold standard (depending on the correlation between the other test and the test being studied). To prevent this type of bias from influencing our estimates of test validity, all patients for whom diagnostic uncertainty exists after history will undergo either surgery or MRA to determine a diagnosis.

## Discussion

The applicability of estimates of specificity and sensitivity are highly dependent on the study design. In terms of evaluating the strength of evidence offered by Phase III studies, there are four general criteria [27]; 1) the sample must be representative of patients for whom clinicians would face diagnostic uncertainty, 2) the results of the diagnostic test cannot influence who undergoes the gold standard, 3) the choice of gold standard must be appropriate, and 4) person’s responsible for interpreting the gold standard and test under evaluation must be unaware of each other’s findings at the time of interpretation.

### Representativeness of the sample

Sackett [1] identified four phases in establishing the validity of a diagnostic tool. A Phase I study asks whether test results in patients with the target disorder differ from those in normal people. A Phase II study asks whether patients with certain test results are more likely to have the target disorder than patients with other test results. Because the diagnosis of patients sampled in Phase I and Phase II studies is known, they provide insight as to whether the particular physical sign shows promise under *ideal* circumstances only. However, the validity of the physical sign cannot be generalized to a real clinical setting in which the patient’s diagnosis is unknown. Unlike Phase I and Phase II studies, Phase III diagnostic studies determine whether the diagnostic test can distinguish among patients with and without the disorder for whom it is clinically reasonable to suspect that the disease may be present. Phase IV studies include research to investigate the effectiveness of a screening program using the diagnostic test and are beyond the scope of this discussion.

Our question provides valuable information about the diagnostic validity of physical examination tests within a clinical setting. Our systematic review of the literature showed that the majority of literature examining the diagnostic validity of shoulder examination tests are Phase I or Phase II studies. The few existing Phase III studies did not meet basic criteria for internal validity and report values of sensitivity and specificity that are likely to be biased; most probably overestimating the true validity of these tests.

Applicability of the results of the study into clinical practice is more likely when the prevalence of disease within the sample represents the prevalence of disease within clinical practice. When the full spectrum of patients for whom the clinician would normally face diagnostic uncertainty are not represented in the study sample the estimates of sensitivity or specificity produced from that study are not valid in the clinical setting. For example, if the sample of study participants includes only those with more severe disease, the study will overestimate the sensitivity of the test since the test is more likely to be positive for these patients. In the same respect, if the sample is composed of individuals who are unlikely to have the disease of interest (i.e. healthy individuals or individuals that clinicians assign a low probability of having the disease of interest), the study will overestimate the specificity of the test since the test is more likely to be negative for these patients.

### Strengths and weaknesses

The strengths of this study include its large sample size, which will enable us to provide precise measures of the specificity and sensitivity of these tests both individually and in combination. In addition, this study involves four surgeons in two different cities in Ontario, Canada, which will increase the applicability of the results to typical tertiary shoulder practices. Since this project is an initiative of surgeons who are members of a large national group there is enormous potential for knowledge transfer in that surgeons across Canada will use the results to guide practice, teach medical students, residents and fellows according to their practice and will create a more research friendly atmosphere with the standardization of tests across Canada.

The limitations of this study include the potential for detection bias since the surgeon who completes the physical examination will also complete the surgical evaluation. We have minimized the potential for this source of bias by creating a standardized protocol for diagnostic shoulder arthroscopy that all surgeons will perform so that all structures are investigated carefully and reported in a standardized fashion. In addition, the time delay between the clinical examination tests and surgical evaluation and the large volume of patients being included in this study reduces the probability that the surgeon will remember the results of the physical examination at the time of surgical evaluation.

By providing strong evidence for the endorsements of some tests over others we increase the likelihood that these endorsements will be adopted into the practice of existing clinicians and become a part of the training of new clinicians. It is also our hope that through adoption of these endorsements, there will be a decrease in the variability between specialists in which maneuvers are used during physical examination and how they are used, all of which will assist in improving the consistency between centers making it easier to conduct research across multiple centers.

## Competing interests

The authors declare that they have no competing interests.

## Authors’ contributions

LS is the principal investigator of the study. DB, KW and LS formed the original study team that developed the research question, wrote the pilot study protocol, obtained local ethics approval, and obtained grant funding. AJ assisted in the development of the statistical plan for this protocol. LS drafted this article, all authors assisted with revisions to the study protocol and methods and approved the final study protocol.

## Pre-publication history

The pre-publication history for this paper can be accessed here:

http://www.biomedcentral.com/1471-2474/14/60/prepub

## Supplementary Material

Additional file 1 Physical Examination Guidelines. Click here for file

Additional file 2 Surgical Evaluation Form. Click here for file
