# Supplementary material for: Protocol for determining the diagnostic validity of physical examination maneuvers for shoulder pathology
Source: BMC Musculoskelet Disord. 2013 Feb 8;14:60. doi: 10.1186/1471-2474-14-60 (PMC3579687; doi:10.1186/1471-2474-14-60)

Physical Examination Guidelines

For use in the research project:

The Diagnostic Validity of Physical Examination Maneuvers for Shoulder Pathology

Strength

External Rotation

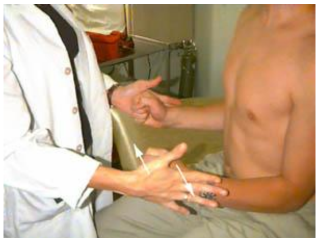

The examiner stands behind the patient. The patient's arms hang down at their side with elbows flexed at 90 degrees, hand and wrist in neutral, midway between full pronation and supination. The examiner reaches from behind patient, each arm pinning an elbow to the patient's side, the examiner's forearm paralleling patient's forearm. The examiner asks the patient to press the back of their wrists or hands outward against the examiner's resistance

Internal Rotation

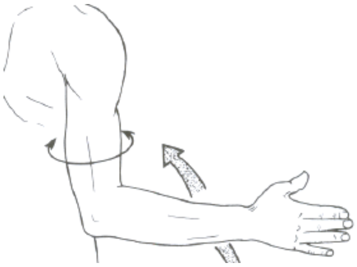

The patient's arms hang down at their side with their elbows flexed at 90 degrees, hand and wrist in neutral, midway between full pronation and supination. The examiner is palm to palm with the patient and asks the patient to press their palms inward against the examiner's resistance

General Rotator Cuff

Transdeltoid Palpation

Palpation is performed anterior to the anterior margin of the acromion through the deltoid. The patient is asked to relax allowing the arm to dangle freely. While positioned behind the patient the examiner holds the patient's forearm with the elbow flexed to allow rotational control in order to maneuver the arm while the examiner's other hand is used for palpation. The arm is gently maneuvered into full extension. Internal and external rotation is used to palpate the rotator cuff tendons. Scoring: In the presence of a tear, both an eminence (prominent greater tuberosity in the presence of a full thickness tear) and a rent 'sulcus' (soft tissue defect created by the rotator cuff that avulsed from the tuberosity) are palpable. The tear is palpated as the arm is brought in and out of full extension and internally and externally rotated.

Supraspinatus

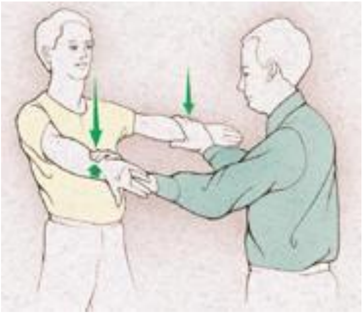

Jobes Supraspinatus Test/Yocum's Test/Empty Can Test

With the patient's arm at 90 degrees of abduction in neutral rotation, the shoulder is then internally rotated and angled forward 30 degrees (scapular plane); the thumb should be pointing towards the floor. The muscle is tested against resistance supplied by the examiner.

Full Can Test
Both of the patient's arms are abducted to 90 degrees in the scapular plane, thumbs up. The examiner places one hand just proximal to each elbow and forcefully presses downward simultaneously. The patient is asked to resist.

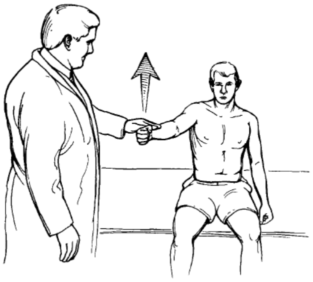

Subscapularis

The Lift Off Test
While standing, the patient places their arm behind their back with the dorsum of the hand resting in the region of the midlumbar spine. The dorsum of the hand is then raised off of the back by maintaining or increasing internal rotation of the humerus and increasing extension of the shoulder. The elbow is kept at a constant flexion angle. To perform this test the patient must have full passive internal rotation and pain cannot be a limiting factor.

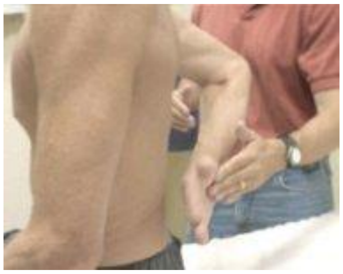

The Belly Press Test
The patient presses the abdomen with the hand flat and attempts to keep the arm in maximum internal rotation.

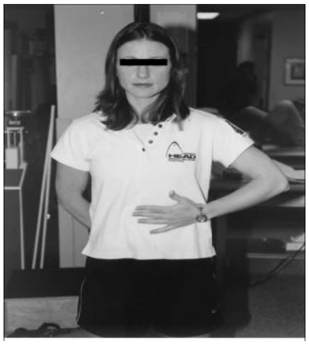

Internal Rotation Lag Sign
The patient is seated with their back to the physician their arm behind their back with the dorsum of the hand resting in the region of the midlumbar spine. The affected arm is held by the physician at almost maximal internal rotation. The elbow is flexed to 90 degrees and the shoulder is held at 20 degrees elevation and 20 degrees extension. The dorsum of the hand is passively lifted away from the lumbar region until almost full internal rotation is achieved. The patient is asked to actively maintain this position as the physician releases the wrist while maintaining support at the elbow.

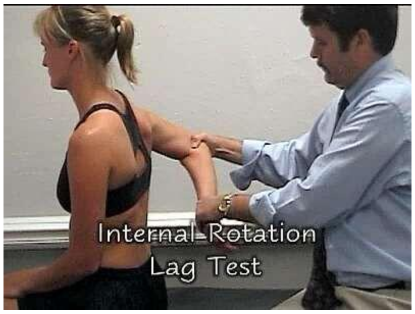

Infraspinatus

Lateral Rotation Lag Sign/External Rotation Lag Sign/Hornblowers Sign
The patient's arm is at their side and the elbow is flexed to 90 degrees. The examiner passively abducts the arm to 90 degrees in the scapular plane, laterally rotates the shoulder to end range and asks the patient to hold the position.

Tendinopathy/Impingement

The Painful Arc
The shoulder is abducted between the angles of 60 to 120 degrees.

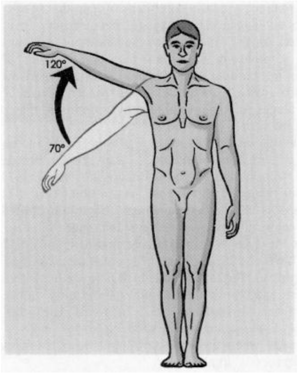

The Hawkins Test
The arm is placed in 90 degrees of forward flexion with the elbow at 90 degrees flexion then the examiners hand, which is grasping the elbow attempts further internal rotation.

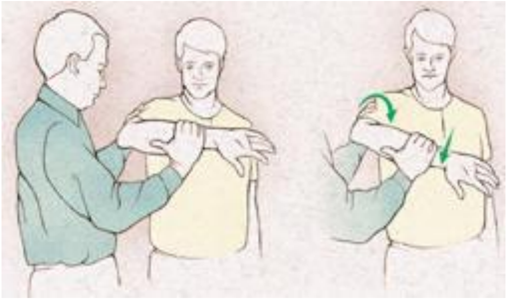

Neer’s Impingement Sign
The scapula is stabilized by the examiner and the arm is forward flexed by the examiner until the patient reported pain or until full elevation was reached.

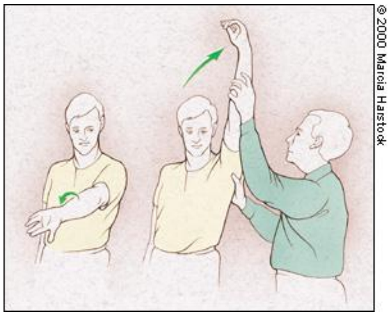

SLAP

Speed's Test

The patient's arm is placed with the forearm in full supination and at 90 degrees of shoulder elevation. The examiner then applies a downward force to the arm and the patient is asked to resist the force.

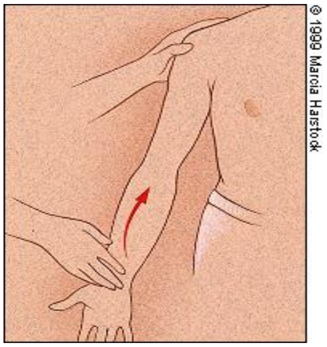

Compression Rotation Test

The patient is in the supine position with the shoulder abducted to 90 degrees and the elbow flexed at 90 degrees. A compression force is applied to the humerus, which is then rotated.

Anterior Slide Test

The patient is examined either standing or sitting with their hands on their hips with thumbs pointing posteriorly. One of the examiners hands is placed across the top of the shoulder from the posterior direction with the last segment of the index finger extending over the anterior aspect of the acromion at the glenohumeral joint. The examiner's other hand is placed behind the elbow and a forward and slightly superiorly directed force is applied to the elbow and upper arm. The patient is asked to push back against the force. Scoring: The test is positive if the patient has pain localized to the front of the shoulder under the examiner's hand and/or a pop or click in the same area or if the manoeuvre reproduces symptoms that occur during their overhead activities

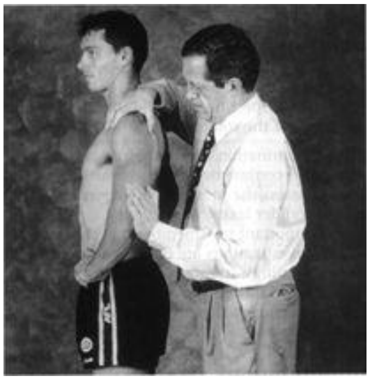

Active Compression Test/O'Briens Test

The examiner stands behind the standing patient and the patient forward flexes the affected arm 90 degrees with the elbow in full extension. The patient then horizontally adducts the arm 10 degrees to 15 degrees medial to the sagittal plane of the body. The arm was then internally rotated so that the thumb is pointing downward. The patient resists as the examiner applies a uniform downward force to the arm. With the arm in the same position the palm is then fully supinated and the maneuver is repeated.

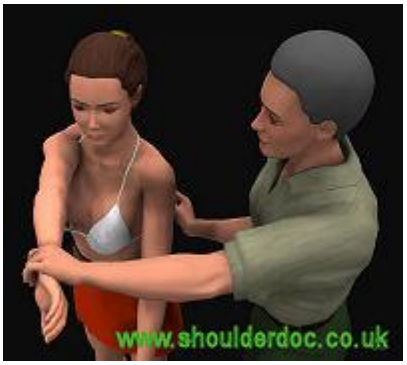

Biceps Load Test I

The patient lies in the supine position. The examiner sits at a right angle and at the same height to the patient on the side of the affected shoulder. The examiner gently grasps the patient's wrist and elbow. The arm is abducted at 90 degrees with the forearm in the supinated position with the elbow flexed to 90 degrees. The patient relaxes and an apprehension test is performed (Taking arm into full external rotation). When the patient becomes apprehensive during the external rotation of the shoulder the external rotation is stopped. The patient is then asked to flex the elbow while the examiner resists (on the same plane as the patients arm so as not to change the degree of abduction and external rotation) the flexion with one hand and asks how the apprehension has changed if at all. The test is repeated and the patient is instructed not to pull the whole upper extremity, just bend the elbow against the resistance.

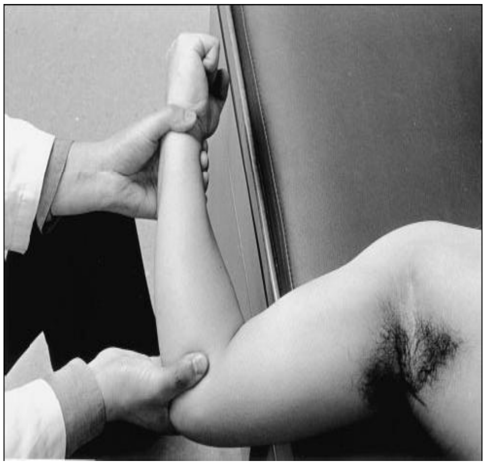

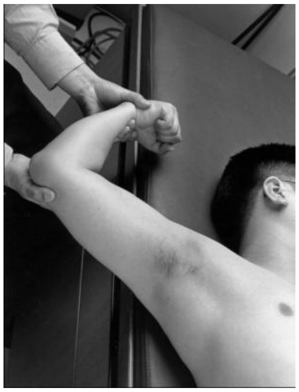

Biceps Load Test II

The patient lies supine. The examiner sits adjacent to the patient on the same side as the affected arm grasping the wrist and elbow gently. The arm is elevated to 120 degrees and externally rotated to its maximal point with the elbow in 90 degrees of flexion and the forearm is supinated. The patient is asked to flex the elbow against the examiner's resistance.

Resisted Supination External Rotation Test

The patient is in the supine position with the scapula near the edge of the table. The examiner stands at the patient's side, supporting the affected arm at the elbow and hand. The arm starts in 90 degrees abduction with the elbow flexed 65 to 70 degrees and the forearm in neutral or slight pronation. The patient is asked to attempt to supinate the hand with maximal effort as the examiner resists and gently externally rotates the shoulder joint to maximal external rotation. The patient is asked to describe the symptoms at maximal external rotation.

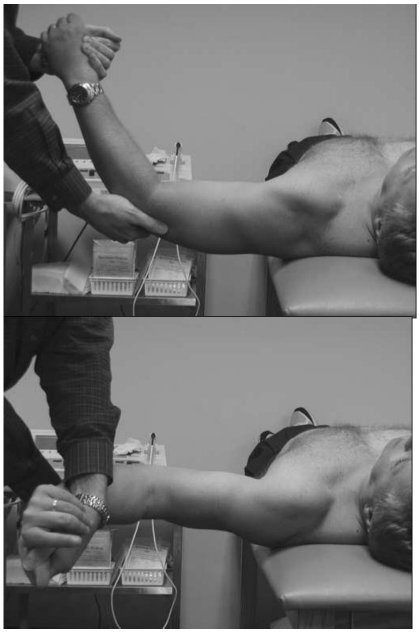

Other Labral Lesions

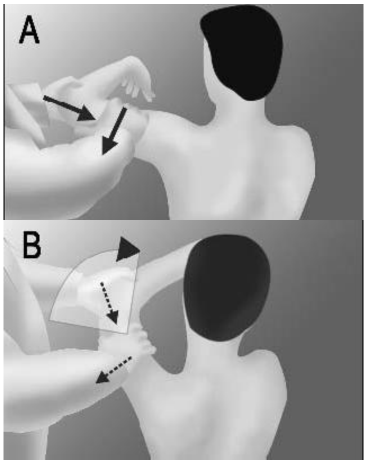

Kim's Test
The patient is in a sitting position against the back of the chair with the arm in 90 degrees of abduction. The examiner holds the patient's elbow and lateral aspect of proximal arm and applies an axial loading force and 45 degrees upward diagonal elevation to the distal arm while inferior and posterior force is applied to the proximal arm.

MDI

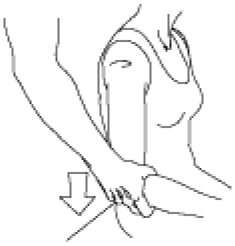

Sulcus Sign
The patient stands with arms hanging at their side. The examiner provides a direct downward traction at the elbow.

Anterior Instability

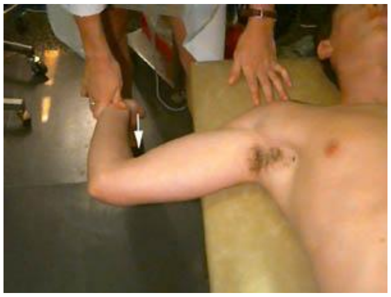

Apprehension Test
With the patient supine and the arm in abduction and external rotation, the examiner pushes anteriorly on the posterior aspect of the humeral head.

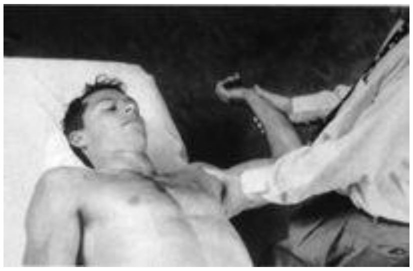

Relocation Test/Fowlers Sign
Following the apprehension test a posteriorly directed force is administered on the humeral head.

Surprise Test/Release Test
While holding the final position of the relocation test, the examiner's hand is quickly removed from the proximal humerus and the patient's response is observed

Load and Shift Test
The patient should be seated. The examiner should be behind the patient on the side to be examined. The examiner places the hand over the shoulder and scapula to steady the limb girdle and then, with the opposite hand, grasps the humeral head. As the head is `loaded' (reduced into the glenoid fossa in a neutral position), both anterior and posterior stresses are applied and the amount of translation is noted. Next, the elbow is grasped and inferior traction is applied. Glenohumeral translation is assessed with the patient supine. Here the arm is grasped in a position of approximately 20 degrees abduction and forward flexion in neutral rotation. The humeral head is loaded and then posterior and anterior stresses are applied

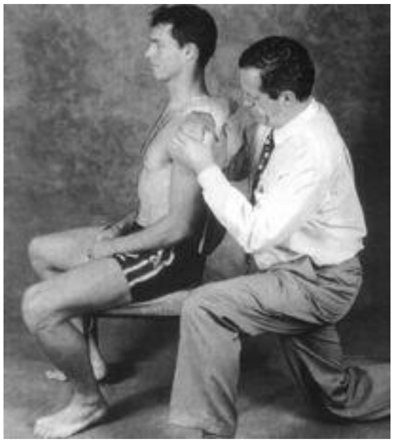

Posterior Instability

Posterior Apprehension/Stress Test
The patient is in the supine or sitting position. The examiner elevates the patient's shoulder in the plane of the scapula to 90 degrees of abduction while stabilizing the scapula with the other hand. While applying the axial load the examiner horizontally adducts and medially rotates the arm. The examiner palpates the head of the humerus with one hand while the other hand pushes the head of the humerus posteriorly. Scoring: The test is positive if the patient is apprehensive or resistant to further motion or if the movement reproduces the patient's symptoms.

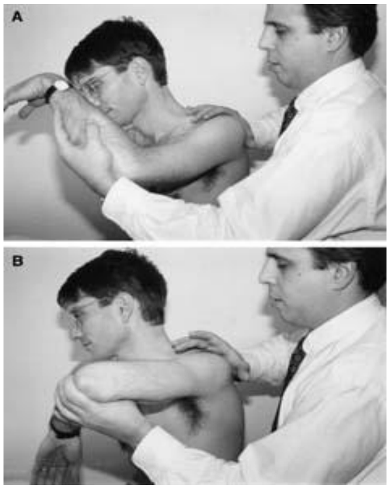

Modified Barlow Test

The patient is in a supine position. The shoulder is in 90 degrees of forward flexion and 0 degrees of rotation. An axial load is placed on the humerus making the humeral head slide posteriorly off the glenoid. The humerus is then abducted and the examiner feels for the humeral head being reduced onto the glenoid.

Acromioclavicular Pathology

O'Briens Test

The patient is asked to stand. The examiner stands behind the patient and the patient forward flexes the affected arm 90 degrees with the elbow in full extension. The patient then horizontally adducts the arm 10 to 15 degrees medial to the sagittal plane of the body. The arm was then internally rotated so that the thumb is pointing downward. The patient resists as the examiner applies a uniform downward force to the arm. With the arm in the same position the palm is then fully supinated and the maneuver is repeated

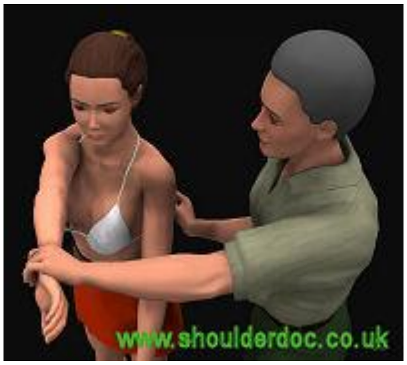

Cross Body Adduction Stress Test

The patient's arm is forward flexed at 90 degrees and then adducted across the body.

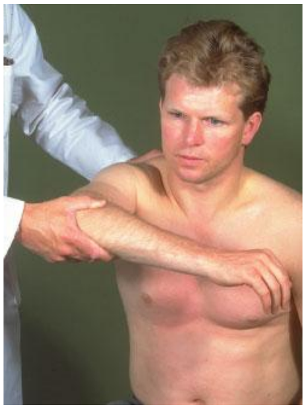

Supplement: Additional file 1 — Physical Examination Guidelines. [file 1471-2474-14-60-S1.pdf]
