# Supplementary material for: Protocol for determining the diagnostic validity of physical examination maneuvers for shoulder pathology
Source: BMC Musculoskelet Disord. 2013 Feb 8;14:60. doi: 10.1186/1471-2474-14-60 (PMC3579687; doi:10.1186/1471-2474-14-60)
Supplement: Additional file 2 — Surgical Evaluation Form. [file 1471-2474-14-60-S2.pdf]

# Shoulder Physical Exam Validity Trial

**Patient ID Number** ☐☐ ☐☐☐☐ (Investigator, Patient #)

**Patient Initials** ☐☐ (F, L)

**Date** ☐☐ ☐☐ ☐☐☐☐ (DD, MM, YYYY)

## Surgical Evaluation

**1. Anterior Labrum**

1.1 Please describe the **anterior labrum** using the following descriptors

- ☐ Normal appearing labrum
- ☐ Degenerative
- ☐ Torn (soft tissue Bankart lesion)
- ☐ Bony Bankart lesion
  - ☐ <10% off of glenoid
  - ☐ 10-25% off of glenoid
  - ☐ >25% off of glenoid

**2. Posterior Labrum**

2.1 Please describe the **posterior labrum** using the following descriptors

- ☐ Normal appearing labrum
- ☐ Degenerative
- ☐ Torn (Reverse Bankart lesion)

.

**3. Cartilage**

3.1 Please describe the **glenoid cartilage** using the following descriptors

- ☐ Normal
- ☐ Grade I (mild cartilage fibrillation)
- ☐ Grade II (significant fibrillation but not to bone)
- ☐ Grade III (fibrillation to bone)
- ☐ Grade IV (exposed subchondral bone)

3.2 If not Normal, please describe the **size** of the lesion

Width ☐☐ - ☐ ☐ cm ☐ mm

Length ☐☐ - ☐ ☐ cm ☐ mm

3.3 Please describe the **humeral head cartilage** using the following descriptors

- ☐ Normal
- ☐ Grade I (mild cartilage fibrillation)
- ☐ Grade II (significant fibrillation but not to bone)
- ☐ Grade III (fibrillation to bone)
- ☐ Grade IV (exposed subchondral bone)

3.4 If not Normal, please describe the **size** of the lesion

Width ☐☐ - ☐ ☐ cm ☐ mm

Length ☐☐ - ☐ ☐ cm ☐ mm

3.5 Please describe whether there is a **Hill Sachs lesion**

- ☐ Present
- ☐ Absent

3.6 If **Present**, please describe the **size** of the lesion

Width ☐☐ - ☐ ☐ cm ☐ mm

Depth ☐☐ - ☐ ☐ cm ☐ mm
